# Supplementary material for: Improving Recognition of Treatable Rare Neuromuscular Disorders in Primary Care: A Pilot Feasibility Study
Source: Children (Basel). 2022 Jul 17;9(7):1063. doi: 10.3390/children9071063 (PMC9317909; doi:10.3390/children9071063)
Supplement: Supplementary file 1 [file children-09-01063-s001.zip › children-1818014-supplementary.pdf]

Supplementary S1 PEDINE Project, Promoting Early Diagnoses In NEuromuscular diseases

Province of Turin (ASL City of Turin, TO3, TO4, TO5, AOU City of Health and Science of Turin)

Step 1; Primary care pediatrician (PCP) recruitment and training phase

Dedicated e-mail to which the PCP has access after formal adhesion to the PEDINE project (adhesion e-mail, subsequent receipt of clinical-teaching material and training proposals, commitment to report patients and the number of balances carried out quarterly); identification for each PCP of the reference NPI with relative contact details.

Step 2: Operational phase: what the PCP does

Identify a Red Flag during health checks in routine activities.

Send an email to the reference NPI with the following: name and surname, red flag number, family contacts and supply to the family the formal documents needed for the specialistic visit.

Step 3: what the reference NPI does

He/She receives the email and makes an appointment within 3-10 days (depending on the type of red flag and therefore the type of delay) communicating it directly to the family. Therefore:

Hypothesis 1: Red flag and evident clinical signs compatible with neuromuscular disease: Immediate sending to the Neuromuscular Reference Center with dedicated email / dedicated contact for direct execution of comprehensive assessment (e.g. blood count, CK, liver and kidney blood function tests, ammonium, lactate, electrolytes, urinary organic acids, plasma amino acids, acylcarnitines, very long chain fatty acids, acid maltase DBS, SMA genetics, DMD genetics, Steinert genetics ...).

Hypothesis 2 Red flag and evident clinical signs compatible with non-NM disease: (e.g. spasticity, neurodevelopmental disorder, genetic syndromes): always CK testing and programming of specific diagnostic/therapeutic path. If CK is high, immediate referral to the Neuromuscular Reference Center with dedicated email / dedicated contact for direct execution of comprehensive assessment.

Hypothesis 3: Red flag confirmed and absence of other clinical signs: protocol neuromuscular tests (CK, SMA genetics, acid maltase DBS); schedule a close inspection in time; if altered tests and/or new signs, immediately referral to the Neuromuscular Reference Center; if negative, clinical monitoring.

Hypothesis 4: Unconfirmed red flag and absence of other clinical signs: send the patient back to the PCP for further monitoring.

Step 4: what the Neuromuscular Reference Center Center does

The center receives the email/contact from NPI and starts a differential diagnostic path for the various neuromuscular diseases

1. If confirmed diagnosis of neuromuscular disease: take in charge the patient as reference center and cooperate with the district NPI service for a specific care and territorial assistance project.

2. If negative findings or if the patient is diagnosed with non-neuromuscular disease: referral to the district NPI service for treatment and clinical monitoring.
